# The role of *Akkermansia muciniphila* in inflammatory bowel disease: Current knowledge and perspectives

**DOI:** 10.3389/fimmu.2022.1089600

**Published:** 2023-01-06

**Authors:** Mengyu Zheng, Ran Han, Yali Yuan, Yunqi Xing, Wenji Zhang, Zhongmei Sun, Yuyue Liu, Junxiang Li, Tangyou Mao

**Affiliations:** ^1^ University of Bristol, Bristol, United Kingdom; ^2^ Dongfang Hospital, Beijing University of Chinese Medicine, Beijing, China; ^3^ Tianjin Nankai Hospital, Tianjin, China

**Keywords:** *Akkermansia muciniphila*, inflammatory bowel disease, barrier function, mucosal immunity, gut microbiota

## Abstract

Inflammatory bowel diseases, including Crohn’s disease and ulcerative colitis, is a chronic relapsing gastrointestinal inflammatory disease mediated by dysregulated immune responses to resident intestinal microbiota. Current conventional approaches including aminosalicylates, corticosteroids, immunosuppressive agents, and biological therapies are focused on reducing intestinal inflammation besides inducing and maintaining disease remission, and managing complications. However, these therapies are not curative and are associated with various limitations, such as drug resistance, low responsiveness and adverse events. Recent accumulated evidence has revealed the involvement of mucin-degrading bacterium *Akkermansia muciniphila* (*A. muciniphila*) in the regulation of host barrier function and immune response, and how reduced intestinal colonisation of probiotic *A. muciniphila* can contribute to the process and development of inflammatory bowel diseases, suggesting that it may be a potential target and promising strategy for the therapy of inflammatory bowel disease. In this review, we summarise the current knowledge of the role of *A. muciniphila* in IBD, especially focusing on the related mechanisms, as well as the strategies based on supplementation with *A. muciniphila*, probiotics and prebiotics, natural diets, drugs, and herbs to promote its colonisation in the gut, and holds promise for *A. muciniphila*-targeted and -based therapies in the treatment of inflammatory bowel disease.

## 1 Introduction

Inflammatory Bowel Disease (IBD), an immunological disorder of the intestinal tract which mainly consists of two clinical phenotypes: ulcerative colitis (UC) and Crohn’s disease (CD), has become a global health problem seriously affecting the quality of life of patients (1, 2). Classic strategies including aminosalicylates, corticosteroids, immunosuppressive agents, and biological therapies to treat IBD are focused on decreasing intestinal mucosal inflammation, inducing and extending disease remission, and managing complications (3, 4). However, these approaches are not curative and are associated with various limitations, such as drug resistance, low responsiveness and adverse events (5). It is, therefore, of fundamental importance to explore the pathogenesis of IBD and develop novel and safe therapeutic strategies.

The intestinal microbiota is a complex and dynamic ecosystem harbouring trillions of microbes that reside in the human intestine and plays a fundamental role in maintaining human health (6). This dynamic interaction between the microbiota and host contributes to the maintenance of the gut epithelial barrier and development and maturation of host immune system (7). Accumulated evidence suggests that intestinal dysbiosis may cause dysregulated mucosal immune responses leading to the onset of IBD in genetically susceptible (8). Therefore, targeted regulation of the composition and function of gut microbiota *via* direct supplement of some specific strains, drug intervention, or dietary structure adjustment plays an indispensable role in maintaining the homoeostatic immune responses and is now considered a valuable therapeutic approach to treat IBD patients. However, how to regulate gut microflora and which specific intestinal strains to regulate have not been clarified. Probiotics such as Lactobacillus or Bacillus cereus have been widely used in the prevention and treatment of IBD. However, a large number of studies show that these probiotics are only used as adjunctive therapy of first-line drugs, and their effectiveness needs to be further evaluated and optimised. Improper usage of probiotics might not only exacerbate existing conditions, but also take advantage of the weak host immunity and cause life-threatening pneumonia, sepsis or gut barrier impairments. A previous review of probiotics revealed that, despite its wide usage, lactobacilli infection poses a considerable threat, with a mortality rate of 48% within one year after infection onset (9). Some strains of lactobacilli such as *L. rhamnousus* have been associated with a high chance of translocation, potentially leading to fatal conditions including human sepsis (10). Thus, the extrapolations of the effects of probiotics need to be carefully scrutinised, and the search for safer, more effective probiotic treatments is urgently needed. Recent studies have revealed the involvement of mucin-degrading bacterium *Akkermansia muciniphila* (*A.muciniphila*) in the regulation of host barrier function and immune response (11), suggesting that it may act as a novel functional microbe with probiotic properties and play an important role in human health and diseases. *A. muciniphila*, a gram-negative bacterium and oval anaerobe which has been proposed as a novel functional microbe with probiotic properties, is initially isolated from human faeces in 2004 (12). However, recent studies have found that *A. muciniphila* is not absolutely anaerobic and can tolerate a small amount of oxygen, as evidenced by its yield and growth rate could increase under micro aerobic conditions (13). As a unique representative strain of the phylum Verrucomicrobia, *A. muciniphila* can be easily detected in phylogenetic and metagenome studies. The abundance of *A. muciniphila* in the human intestinal tract represents 1-4% of the total faecal microbiota, and will change with age, diet, body mass and immune state during a lifetime (14, 15). *A. muciniphila* is known to colonise the mucosal layer of the human intestine by utilising the glycoprotein mucin as its direct source of carbon and nitrogen (16), which means it has a unique survival advantage, that is, it can still survive during nutrient deficiency and under harsh conditions (17). With the characteristics of mucosal degradation, *A. muciniphila* has the function of regulating the intestinal barrier and immune response, and plays an important role in human health and diseases. Reduced levels of *A. muciniphila* have been observed in patients with IBD, indicating that it may contribute to the process and development of inflammatory bowel diseases, and maybe a potential target and promising strategy for the therapy of inflammatory bowel disease (18). However, the exact role and specific mechanisms of *A. muciniphila* in inflammatory bowel disease require further investigation.

Here, we reviewed the latest research and summarised the current knowledge of the role of *A. muciniphila* in IBD. This review reveals an interesting phenomenon that not only viable *A. muciniphila*, but also pasteurised *A. muciniphila*, and its ingredients such as AmEVs, Amuc_1100 and P9 also can alleviate intestinal inflammation, suggesting a therapeutic potential of *A. muciniphila* in the treatment of IBD. The protective role of *A. muciniphila* and its ingredients in IBD mainly involves immune regulation and enhanced barrier function, modulation of gut dysbiosis and colonisation against other pathogens. Moreover, we summarised the strategies based on supplementation with *A. muciniphila*, probiotics and prebiotics, natural diets, drugs, and herbs to promote its colonisation in the gut, and hold promise for *A. muciniphila*-targeted and -based therapies in the treatment of IBD.

## 2 Relationship between *A. muciniphila* and inflammatory bowel disease

### 2.1 Insights from clinical studies

Accumulated evidence has revealed a significant reduction in *A. muciniphila* in both faecal samples and mucosal biopsies of patients with IBD. The research from Png et al. showed a significant increase in the total abundance of mucosa-associated bacteria, while the numbers of *A. muciniphila* were reduced by mean 92-fold and 172-fold in noninflamed and inflamed UC, respectively, and 14.8-fold in inflamed CD, suggesting that *A. muciniphila* may have a potential protective or anti-inflammatory role in health (19). Earley et al. quantitatively analysed the amount of *A. muciniphila* in mucus brushings of the colonic mucus. They found a significant reduction in all four areas of the colon, caecum, transverse colon, left colon and rectum from patients with active UC compared to quiescent UC and healthy controls. In addition, the abundance in quiescent UC returns towards levels observed in the healthy colon, which strongly demonstrates an inverse relationship between *A. muciniphila* and intestinal inflammation (18). They further revealed a positive association between *A. muciniphila* abundance and the percentage of sulphated mucin in the mucus gel layer. James et al. collected the total faecal output of patients with UC in remission, and assessed faecal microbial abundances by quantitative PCR. The result showed that faeces from patients with UC had more diverse microbiota in their *Clostridium cluster XIVa* and a lower proportion of *A. muciniphila* (20). L.K. Vigsnæs et al. investigated the alterations in the composition of the gram-negative bacterial population in patients either with UC in remission or with active disease, and in healthy controls and found a significantly lower density of *A. muciniphila* in UC patients with active disease, but no significant difference between the healthy controls and the UC patients in remission, suggesting a possibility that reduction of *A. muciniphila* may play a role in the development of UC (21). Rajilić-Stojanović et al. also found that faeces of patients with UC in remission showed a significant reduction (approximately 5-fold) in the abundance of *A. muciniphila* (22). Wang L et al. comfirmed that *A. muciniphila* in stool samples was significantly reduced in patients with UC (23). Paediatric studies were mostly consistent with the above findings, and Dunn et al. reported that CD patients aged 10 to 16 had lower microbial diversity than the healthy controls, and the abundance of *A. muciniphila* was lower in patients who did not achieve sustained remission than those who did (24).

However, there are also a few studies that showed contradictory results. Lopez-Siles and colleagues found no difference in *A. muciniphila* across different disease states, whether it is UC, CD or colorectal cancer (CRC), although CD patients whose disease onset were below 16 years of age observed a marked depletion of this species (25). Moreover, the relative representation of *A. muciniphila* was found to increase in CD patients according to Danilova et al. (26). Thus, the role of *A. muciniphila* in IBD still requires further investigations. **Table 1** demonstrates the overview of clinical studies of *A. muciniphila* related to inflammatory bowel disease.

**Table 1:** Overview of clinical studies of *A. muciniphila* related to inflammatory bowel disease.

Object of study	Publication year	Cohort description	Sample type	Outcome characteristics	Microbiota analysis approach	References
Adult	2010	20 UC, 26 CD, and 20 controls	Mucosal biopsies	The numbers of *A. muciniphila* were reduced	Quantitative PCR	Png et al.
Adult	2012	6 UC in remission, 6 UC in active and 6 controls	Faecal samples	The average density of *A. muciniphila* in active UC patients was significantly lower than that in the healthy controls	Quantitative PCR	Vigsnæs et al.
Adult	2013	15 UC in remission and 15 controls	Faecal samples	A significant reduction in the abundance of *A. muciniphila* was observed	HITChip Phylogenetic Microarray Analysis	Rajilić-Stojanović et al.
Adult	2014	19 UC in remission and 10 controls	Faecal samples	Faeces from the UC cohort had lower proportions of *A. muciniphila*	Quantitative PCR	James et al.
Adult	2018	17 controls, 23 UC, 31 CD, 3 IBS and 3 CRC	Colonic biopsies	A marked depletion of *A. muciniphila* was observed in CD patients with disease onset below 16 years of age.	16S rRNA sequencing, quantitative PCR	Lopez-Siles et al.
Adult	2019	20 active UC, 14 quiescent UC and 20 controls	Colonic biopsies and mucus gel layer brushing	Patients with active UC had reduced abundance of *A. muciniphila*, and there is an inverse relationship between *A. muciniphila* and intestinal infammation	Quantitative PCR	Helen eEarley et al.
Adult	2019	78 UC, 17 CD, 96 controls	Faecal samples	*A. muciniphila* was increased in CD patients	Whole-genome sequencing	Danilova et al.
Adult	2020	58 active UC, and 72 controls	Faecal samples	The abundance of *A. muciniphila* was significantly decreased in patients with IBD	16S rRNA sequencing, Quantitative PCR	Wang et al.
Paediatric	2016	10 CD patients (age 10-16), 5 healthy controls (age 9-14)	Faecal samples	Microbial diversity was lower in Crohn’s disease patients comparing to controls and lowest in patients who did not achieve sustained remission.	16s rRNA sequencing	Dunn et al.

### 2.2 Insights from animal studies

Similar to human clinical subjects, preclinical reports have also shown that the amount of *A. muciniphila* from animals with experimental colitis is negatively correlated with the severity of the disease. Previous studies showed that the abundance of *A. muciniphila* was significantly decreased in experimental colitis mice. Alrafas et al. investigated the gut microbial composition in BALB/c mice with 2,4,6-trinitrobenzene sulfonic acid (TNBS) -induced colitis by analysing the caecal flush from these mice (27). They reported that *Bacteroides acidifaciens*, a species that could lead to the aggravation and development of colitis, has significantly increased in colitis, whereas *A. muciniphila* has markedly decreased. The reduction in *A. muciniphila* suggested its possible role in maintaining gut homeostasis and that the restoration of this species may contribute to alleviating microbial dysbiosis due to IBD. Wang and colleagues agreed with the above conclusion, and they analysed the faecal samples of C57BL/6 mice with DSS-induced colitis, and although the animal model was different, they also found that the abundance of *A. muciniphila* experienced a marked decreased in colitis (24). Mice with chronic restraint stress and DSS collectively induced colitis also showed marked a decrease in *A. muciniphila* but an increase in the proportion of Parabacteroides, Erysipelatoclostridium and Enterococcus, suggesting a dysbiosis in gut microbiota after disease induction (28). Studies on epithelium-specific autophagy-related 5 (Atg5) knockout mice, a strain that is innately susceptible to colitis, were also consistent with the aforementioned animal or clinical studies: inflammation-controlling *A. muciniphila* was reduced, while pro-inflammatory bacteria (e.g., *Candidatus Arthromitus*) and potential pathogens observed an increase (29).

However, a small number of mice studies, especially those focusing on mice with genetic mutations, have shown inconsistent or even contradictory results. Mahoro et al. did not observe any significant difference in *A. muciniphila* abundance between SD rats with or without DSS treatment (30), while colonic mucosal samples from female C57BL/6 mice with DSS-induced colitis even showed higher *A. muciniphila* abundance than their counterparts in the control group according to Håkansson’s team (31). Furthermore, investigations on the faecal samples from two genetically colitis-prone mice, namely IL10^-/-^ and Winnie^-/-^ mice, both witnessed more abundant *A. muciniphila* than their wild-type littermates (32, 33). Cyp27b1^-/-^ mice with 1,25(OH)_2_D_3_ deficiency is another colitis-prone strain that develops colonic inflammation at the age of 8-10 month. Zhu et al. confirmed that *A. muciniphila* was significantly enriched in Cyp27b1^-/-^ mice, and supplementation with 1,25(OH)_2_D_3_ could reduce *A. muciniphila* abundance. Interestingly, while this results from experiment indicate that 1,25(OH)_2_D_3_ limits the colonisation of A. muciniphila, vitamin D deficiency has been shown to reduce the level of *A. muciniphila* in wild type BALB/c and C57BL/6 mice fed with high-fat diet (34, 35). The authors suggest that this discrepancy might be due to difference in mouse models (36), suggesting that genotypes have to be taken into consideration when studying the role of *A. muciniphila* in health and in disease states.

It should be noted that the strain-specific properties of *A. muciniphila* should also be taken into consideration. Zhai et al. investigated and compared the characteristics of *A. mucinipihila* type strain ATCC BAA-835 and murine *A. muciniphiola* strain 139 and reported interesting results. Both strains exhibited anti-inflammatory effects including inhibiting IL-8 production by TNF-α-stimulated, co-cultured HT-29 cells in in-vitro anti-inflammatory test. However, these two strains functioned differently in DSS-induced colitis mice models. While administrations of both strains alleviated colitis symptoms by downregulating pro-inflammatory cytokines, only the DSS+ATCC group observed a significant reduction of spleen weight and inflammation index in comparison to the DSS group. DSS+139 group. In addition, the DSS+ATCC group was the only group whose histological score recovered to the level of the healthy control group. On the other hand, while recovery from chronic colitis was also accelerated in the DSS+139 group, the results were not significant (37). Overview of animal studies of *A. muciniphila* related to inflammatory bowel disease is shown in **Table 2**.

**Table 2:** Overview of animal studies of *A. muciniphila* related to inflammatory bowel disease.

Publication year	Object of study	Cohort description	Sample type	Outcome characteristics	Microbiota analysis approach	References
2013	C57BL/6 mice	5 days of DSS administration was used to induce colitis.	Faecal samples	Administration of DSS decreased the composition of AmEVs in mice stool samples.	16s rRNA sequencing	Kang et al.
2015	Female C57BL/6 mice	Mice were given DSS in drinking water for 7 days to induce colitis.	Colonic mucosal samples	The amount of *Akkermansia* was significantly higher in the DSS group.	16s rRNA sequencing, quantitative PCR	Å. Håkansson et al.
2017	IL10-/- mice	Colitis-prone mice (IL10-/-).	Faecal sample	*A. muciniphila* was increased in colitis mice.	16S rRNA sequencing, quantitative PCR	Seregin et al.
2018	C57BL/6J epithelium-specific autophagy-related 5 (Atg5) knockout mice	Faecal samples of C57BL/6J Atg5−/− mice were collected for bacterial composition analysis.	Faecal samples	*A. muciniphila* was decreased, whereas pro-inflammatory bacteria and potential pathogens were increased.	16s rRNA sequencing	Yang et al.
2019	BALB/c mice	Mice were given intrarectal administration of 1 mg TNBS in 0.1 ml of 50% ethanol	Caecal flush	The abundance of *A. muciniphila* was decreased in TNBS-induced colitis mice.	16s rRNA sequencing	Alrafas et al.
2019	C57BL/6 mice	Male C57BL/6 mice. Were administered 2% (wt/vol) DSS from day 0-6.	Faecal samples	The abundance of *A. muciniphila* was decreased after disease induction.	16s rRNA sequencing	Bian et al.
2019	C57BL/6J mice	8 days of 2% DSS-containing water were given to induce colitis.	Faecal samples	The abundance of *A. muciniphila* was decreased in mice with colitis.	16s rRNA sequencing, quantitative PCR	Wang et al.
2019	*Winnie*-/- mice	Colitis-prone mice (Winnie-/- mice with Muc2 mutation).	Faecal samples	*A. muciniphila* was more abundant in Winnie mice than their wild type littermates.	16s rRNA sequencing, quantitative PCR	Liso et al.
2019	Cyp27b1^−/−^ mice	Colitis-prone mice (Cyp27b1^−/−^ with 1,25(OH)_2_D_3_ deficiency)	Faecal samples	The level of *A. muciniphila* significantly increased after colitis onset	16s rRNA sequencing	Zhu et al.
2021	C57BL/6N mice	Chronic restraint stress (limiting mice in 50ml tube for 30 days) followed by 7 days of 2.5% DSS administration induced colitis in mice	Faecal samples	The abundance of *A. muciniphila* was decreased after disease induction.	16s rRNA sequencing, quantitative PCR	Gu et al.
2021	SD rats	3% DSS was orally administered for 7 days, followed by a 7-day recovery phase	Faecal sample	No significant difference in *A. muciniphila.*	16s rRNA sequencing	Mahoro et al.

## 3 The role of *A. muciniphila* in inflammatory bowel disease

### 3.1 Immunomodulatory properties

Gut microbial structure can regulate host metabolism and homeostasis by influencing the immune system, thereby regulating intestinal endocrine system and preventing pathogen overgrowth. Previous studies suggested that *A. muciniphila* can reduce the level of colon infiltrating macrophages and cytotoxic T lymphocytes (CTLs) to alleviate colitis (24), but increase the number of anti-inflammatory regulatory T (Treg) cells in high-fat diet-fed mice (38). The increase in Tregs may be due to the elevation in short-chain fatty acids (SCFAs; e.g., acetate, propionate, iso-buturate, iso-valeric and valeric) in the host gut following promoted *A. muciniphila*, which activates G protein-coupled receptor 43 (GPR43) and GPR41, thereby promoting Foxp3+Tregs in the host colon, relieving colitis and changing the composition of the gut microbiota (39). The conversion from CD4+T cells (CD3+CD4+) to Foxp3+ Treg was also promoted in mice treated with *A. muciniphila* (37). While the proportion of dendritic cells infiltrating mLNs did not significantly change, the mean fluorescence intensity of antigen-presenting molecule MHC-II on CD11b-CD103+ dendritic cell subpopulation was reduced in their study, indicating a decrease in activities of these subpopulations. Katiraei et al. further explored the immunomodulatory effects of *A. muciniphila* in mice. They revealed that *A. muciniphila* increased the total B cell population without changing the percentage of follicular or mucosal B cells in mesenteric lymph nodes (mLN), whereas total T cell and total neutrophil populations observed a marked decrease (40). Ansaldo et al. also studied the intestinal adaptive immune response induced by *A. muciniphila* and demonstrated that *A. muciniphila* triggers the production of IgG1 antibodies and induces antigen-specific T cell responses in mice, promoting the differentiation from naïve T cells to T follicular helper cells (41).

Interestingly, both pasteurised *A. muciniphila* and Amuc_1100, a specific outer membrane protein of the bacterium, relieved colitis symptoms, including colon shortening, colon injuries, inflammatory cell infiltration and impaired barrier functions. Flow cytometry analysis supported these findings, showing pronounced downregulation of pro-inflammatory cytokines such as TNF-α, IFN-γ, IL-1β, IL-6, IL-18 and IL-33. Specifically, CD16/32^+^ macrophages and CD8+ CTLs in the colon, as well as the level of CD16/32^+^ macrophages in the spleen and mesenteric lymph nodes were reduced in colitis mice (23). Hippala et al. reported that *A. muciniphila* increased the release of anti-inflammatory cytokine IL-10, although it also induces a low-level pro-inflammatory IL-8, which may be beneficial as it keeps the host immune system alerted (42). Ottman et al. backed their results by revealing that Amuc_1100 can activate Toll-like receptor2, which then leads to the production of IL-10 (43). Beyond improving colitis, Amuc_1100 also triggers immune responses that delay tumour formation and inhibit tumour growth. γH2AX or Ki67 expression were both reduced after pasteurised *A. muciniphila* or Amuc_1100 administration, indicating that these treatments may attenuate double-stranded DNA breaks and inhibit cell proliferation, respectively (23).

However, *A. muciniphila* has also been reported to induce colitis in germ-free IL10-deficient mice, indicating that *A. muciniphila* is not an elixir for IBD under all circumstances. *A. muciniphila* caused increased bacterial translocation, while both viable and heat-killed increased the production of cytokines (IL-6 and IL-12p40) of IL10^-/-^ mice bone-marrow-derived macrophages. Further analysis suggests that the immunostimulatory effect of *A. muciniphila* in these genetically susceptible mice was not due to its proteins or nucleic acid, but its polysaccharide. Therefore, potential *A. muciniphila*-based therapy should only be taken after evaluating the genotypes and disease states of the subject (32).

### 3.2 Intestinal mucosal barrier function

Mucus layer thickness is an essential measure of intestinal permeability that is decreased in diet-induced obesity and IBD. Secreted by the goblet cells, intestinal mucus consists of about 20% protein and 80% carbohydrates, making it a substrate and source of nutrients for *A. muciniphila*. Gut mucus can be further divided into two sections, an inner layer with the absence of bacteria, and a thicker layer inhabited by commensal bacteria (44). The mucus forms a physical barrier between the gut and noxious agents and microorganisms, preventing them from directly contacting the epithelial cells and potentially entering the circulation. Thinner mucus layers and increased gut permeability lead to the translocation of lipopolysaccharide (LPS) from the intestine to circulation, consequently resulting inflammation and metabolic endotoxemia. A number of studies have shown that *A. muciniphila* can improve mucus thickness and thus gut barrier integrity. Our previous study revealed that *A. muciniphila* colonisation in mice promotes goblet cells, which were significantly reduced due to *C. rodentium* infection-induced colitis (45). Everard et al. showed that oral administration of viable or pasteurised *A. muciniphila* could significantly increase the population of mucin-secreting goblet cells, thereby restoring the 46% loss of mucus during obesity (46). Shin et al. proposed that increased goblet cells was resulted from *A. muciniphila* stimulating mucus turnover rate (38). As *A. muciniphila* degrades mucin into SCFAs, goblet cells’ preferable source of energy, mucin synthesis can be significantly boosted. Conversely, the amount of mucin can influence the abundance of *A. muciniphila*. Earley et al. hypothesised that the reduced abundance of *A. muciniphila*, as observed in UC patients, is resulted from a lack of sulphomucin, a substrate of *A. muciniphila*. Their hypothesis was supported by the observed association between inflamed mucosa and reduced sulphomucin in acute UC patients (18). However, the lack of sulphomucin is unlikely the sole contributor, as altered glycosylation and reduced MUC2 mucin may also have an impact on microbial survival (47). In line with these observations, the results of a previous study from our team also showed that colonisation of mice with *A. muciniphila* displayed markedly increased numbers of goblet cells in colons, resulted in upregulated expressions of gene encoding mucin, including muc1, muc5, and muc13. These results suggest a protective effect of *A. muciniphila* on colitis involved regulation of the mucus barrier in the gut (46).

Another study on *A. muciniphila* extracellular vesicles (AmEVs), a bilayer structure composed of lipid, protein, lipopolysaccharides as well as other molecules, showed the ability to regulate intestinal barrier permeability by altering tight junction protein expression. The three major tight junction proteins responsible for maintaining barrier integrity, namely occludin, zonal occludins and claudin-5, were promoted after AmEVs administration, thus alleviating the reduction of these proteins during high fat diet (HFD) treatment (48). This improvement is likely to result from Amuc_1100 activating TLR2, a receptor responsible for regulating a wide range of tight junction proteins including occludin (49). Their conclusion is evidenced by *in vitro* cell cultures: Caco-2 cell barrier integrity was improved by AmEVs *via* AMPK activation, which in turn may be promoted by increasing SCFA levels as suggested by previous studies (50). Li et al. also reported that occludin and ZO-1 were up-regulated in Apoe-/- mice as *A. muciniphila* prevented the Western diet- induced inflammation and endotoxemia (51).

### 3.3 Modulation of gut dysbiosis and colonisation against other pathogens

A number of studies have showed that administrations of *A. muciniphila* can influence the diversity and number of gut microbiota, which are altered in IBD. Suspected pathogenic bacterial agents for IBD include *Escherichia coli* and the *Helicobacter species*, and IBD is also accompanied by reductions of beneficial species, such as *Lactobacillus*, *Bifidobacterium* and *A. muciniphila* itself (52, 53). Bian et al. reported a positive correlation between *A. muciniphila* administration upregulated cytokine IL-10, which in turn and was positively correlated with a number of species that was promoted by *A. muciniphila* (e.g., *Verrucomicrobia*, *Akkermansia* and *Ruminococcaceae*) (54). On the other hand, *Bacteroidetes*, a harmful species associated with colitis progression was reduced by *A. muciniphila* administration.

**Figure 1:**
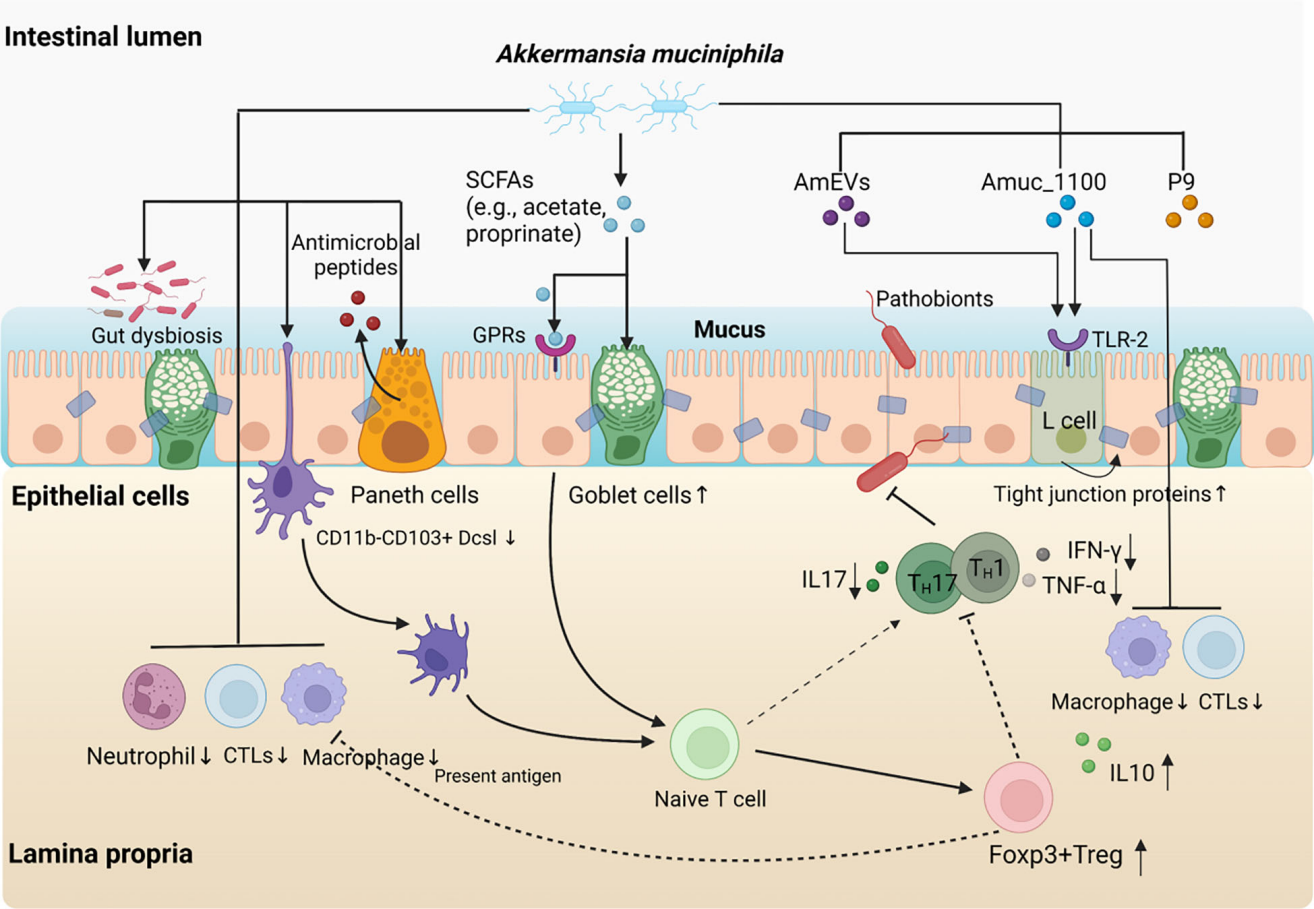
The role of *A. muciniphila* in inflammatory bowel disease. Supplement with *A. muciniphila* reduces the levels of colonic infiltrating macrophages and cytotoxic T lymphocytes (CTLs) and relieves intestinal inflammation. The protective effects of *A. muciniphila* on intestinal homeostasis are associated with increased numbers of goblet cells, enhanced mucus barrier and anti-microbial responses. *A. muciniphila* supplement partially attenuates gut dysbiosis and resist the colonisation against other pathogens. Moreover, *A. muciniphila* derived SCFAs promoted the conversion from Naïve T cells to Foxp3+Treg cells through GPRs signal pathway, which may be accompanied by reduced Th1/17 cell-mediated inflammatory response. Interestingly, both pasteurised *A. muciniphila* and Amuc_1100, a specific outer membrane protein of the bacterium also play a similar role as *A. muciniphila* itself in immune protection and inhibit the infiltration macrophages and CTLs. Furthermore, *A. muciniphila*-derived extracellular vesicles (AmEVs), a bilayer structure composed of lipid, protein, lipopolysaccharides as well as other molecules, and Amuc_1100 also regulate intestinal barrier permeability by altering tight junction protein expression through activating TLR2 pathway.

In addition to promoting preferred bacteria, *A. muciniphila* may also inhibit the harmful effects of unwanted bacteria in the colon. Recent analysis of *A. muciniphila* genome suggested that *A. muciniphila* might produce cysteine by utilising hydrogen sulphide. Hydrogen sulphide is the end product of sulphate-reducing bacteria, and both are involved in the pathogenesis of chronic bowel diseases including UC (55). Hydrogen sulphide is also a potent genotoxin that may be associated with colorectal cancer (56). With *A. muciniphila* consuming hydrogen sulphide, it may partially limit the toxicity of sulphate-reducing bacteria to the host (42). Moreover, *A. muciniphila* augmented the expression of RegIIIγ, the primary anti-microbial peptides produced by Paneth cells. RegIIIγ, along with other anti-microbial peptides, participate in maintaining the innate immunity of the gut barrier as well as the gut microbiota composition (47). qRT-PCR analysis from our previous studies further evidenced the promoting effect of *A. muciniphila* on antimicrobial peptides: RegIIIγ and CRAMP were both upregulated, and both of which contributes to the protection against pathobionts infection. *A. muciniphila* also increased IL-22 expression, which has been suggested to augment mucus and antimicrobial proteins expression and subsequently facilitate the dissemination of pathogenic bacteria in the host gut (46). The role of *A. muciniphila* in IBD is shown in **Figure 1**.

## 4 Strategies to promote abundance of *Akkermansia muciniphila*

Given that *A. muciniphila* is negatively correlated with IBD and plays an important role in maintaining homeostasis of intestinal barrier and mucosal immunity, we believe that the elevated abundance of *A. muciniphila* in the gut microenvironment *via* direct supplement of *A. muciniphila* and its components, indirect interventions such as derivatives, drugs, dietary or herbs may be a novel and promising strategies for the treatment of IBD. Here, we summarised these strategies, discussed their potential as therapeutic reagents, and holds promise for *A. muciniphila*-targeted and -based therapies in the treatment of IBD (**Tables 3**, **4**).

**Table 3:** Pre-clinical studies on the administration of the *A. muciniphila* and its components for protection against inflammatory bowel disease.

Publication year	Bacterial strains	Subject	Daily dose	Duration	Outcome characteristics	References
2010	*A. muciniphila* MucT (ATTC BAA-835)	Male C57BL/6 mice was administered *A. muciniphila* followed by 7 days DSS treatment.	3 × 10^9^ CFU	14 days	*A. muciniphila* ameliorated mucosal inflammation *via* protecting the gut barrier function and reducing inflammatory cytokines, and improving the microbial community	Chin Wen Png et al.
2016		*Apoe−/−* mice were fed Western diet for 8 weeks and lipopolysaccharide was subcutaneously administered for the last 4 weeks. *A muciniphila* was then orally gavaged for 8 weeks.	5×10^9^ CFU	16 weeks	*A. muciniphila* decreased intestinal permeability and reduced systemic inflammation	Li et al.
2019		Male C57BL/6 mice were administered 2% (wt/vol) DSS from day 0-6 and were administered *A. muciniphila* from day -7 – day 7.	3 × 10^9^ CFU	14 days	*A. muciniphila* was increased and colitis symptoms were alleviated	Bian et al.
2020		Male E3L.CETP mice were given Western diet for 7 weeks, during which mice received oral gavage of *A. muciniphila* for the last 4 weeks.	2 × 10^8^ CFU	7 weeks	*A. muciniphila* increased the total B cell population and decreased the total neutrophil and T cell population in the mesenteric lymph node. Dendritic cell activities were also reduced.	Katiraei et al.
2021		Female BALB/c mice received *A. muciniphila* oral gavage for one week after being depleted all gut microbiota with antibiotic cocktail pre-treatment. *C. rodentium* was then used to induce colitis.	1 × 10^8^ CFU	19 days	*A. muciniphila* attenuated C. *Citrobacter rodentium*-induced colitis	Mao et al.
2022		CaCl_2_ was used to induce abdominal aortic aneurysms, and mice were given *A. muciniphila* by oral gavage.	2 × 10^8^ CFU	28 days	*A. muciniphila* increased the number of gut microbiota and α diversity in AAA mice. Pro-inflammatory cytokines such as IL-33 were restored.	He et al.
2017	Viable and heat-killed *A. muciniphila*	Il10/mice were gavaged with *A. muciniphila* or B. acidifaciens.	2 × 10^8^ CFUs	8 weeks	Both viable and heat-killed *A. muciniphila* promoted IL-6 and IL-12p40 production of IL10-/- mice bone-marrow derived macrophages, and *A. muciniphila* induced bacterial translocation and upregulated cytokines including IL-6 and IL-12p40	Seregin et al.
2013	*A. muciniphila* (ATCC BAA-835) extracellular vesicles (AmEVs)	C57BL/6 mice were given water containing DSS and AmEVs for 5 days.	AmEVs: 100 mg	8 days	Mice experiment showed that AmEVs alleviated DSS-induced IBD	Kang et al.
2018		C57BL/6 mice were fed a 60% HFD for 12 w before given oral administration of *A. muciniphila* extracellular vesicles (AmEVs).	AmEVs: 10 μg	14 weeks	AmEVs improve permeability by regulating tight junctions	Chelakkot et al.
2020	Pasteurised *A. muciniphila* MucT (ATCC BAA-835), and Amuc_1100	Male C57BL/6J mice were given oral administration of pasteruised *A. muciniphila* or the protein Amuc_1100 from 2 weeks before DSS treatment to sacrifice.	Pasteurised *A. muciniphila*:1.5×108 CFU; Amuc_1100:3 μg	22 days	Amuc_1100 supplementation increased the abundance of *A. muciniphila* in colitis mice	Wang et al.
2021	P9 from *A. muciniphila* (ATCC-BAA-835)	HFD-fed C57BL/6J were orally administered P9 for 8 weeks.	P9: 100μg	8 weeks	Anti-inflammatory M2 macrophages (CD11b+CD206+) was higher in mice administered with *A. muciniphila*	Yoon et al.

**Table 4:** Strategies to promote abundance of A. muciniphila in colitis animal model.

Strategy	Publication year	Intervention	Subject of study	Sample type	Outcome characteristics	Microbiota analysis approach	References
Probiotics	2015	Mice were given 3% w/v DSS from day 0-6 before being administered Lactobacillus *rhamnosus* SHA113 from day 7 - day 17.	*Lactobacillus rhamnosus (*10^9^ CFU/ml)	Male C57BL/6Cnc mice	UC was attenuated in mice and *A. muciniphila* abundance was increased.	16S rRNA sequencing, quantitative PCR	Pang et al.
	2016	Mice were given daily oral administration of *Lactobacillus rhamnosus, Bifidobacterium animalis subsp. lactis* or Mix for 1 week. Treated mice were then fed HFD for 7 or 16 weeks	*Lactobacillus rhamnosus* LMG S-28148 (30 μl, 10^9^ CFU), *Bifidobacterium animalis subsp. lactis* LMG P-28149 (30 μl, 10^9^ CFU) or Mix (30 μl, 10^8^ CFU of each strain)	C57BL/6J	*Bifidobacterium animalis subsp. lactis* and Mix-treated mice observed an increased level of *A. muciniphila*, while *Lactobacillus rhamnosus-*treated mice observed a decrease	Quantitative PCR	Alard et al.
	2019	Mice with DSS-induced colitis were treated with quadruple probiotics.	*B. infantis, L. acidophilus, E. faecalis* (1.5 × 10^9^ CFU respectively) and B. cereus (0.5 × 10^8^ CFU)	Male C57BL/6 mice	Microbiota structure (including *Akkermansia*) was almost restored to normal.	16S rRNA sequencing, quantitative PCR	Chen et al.
	2020	*L. pentosus* was intragastrically administered to mice after colitis induction for 7 days.	*Lactobacillus pentosus (*5 _ 10^9^ CFU/mL)	ICR mice	*L. pentosus* increased *A. muciniphila* abundance.	16S rRNA sequencing, quantitative PCR	Ma et al.
	2020	Male triathletes were given *Lactobacillus plantarum* PS128 for 4 weeks	*Lactobacillus plantarum* PS128 (3 × 10^10^ CFU/day)	Male triathletes	Modulations in gut microbiota of participants were observed, including significant increases in *Akkermansia*, *Bifidobacterium* and *Lactobacillus*	16S rRNA sequencing	Huang et al.
Prebiotics	2011	Male C57BL/6J mice were given 2.5% DSS for colitis induction before supplemented with TFA by intragastric gavage for 7 days	TFA (125mg/kg or 62.5mg/kg)	Male C57BL/6 J mice	TFA improved DSS-induced experimental colitis and increased *A. muciniphila* abundance.	16S rRNA sequencing, quantitative PCR	Bu et al.
	2015	Mice were fed HFD or HFD containing soy protein isolate-sorbed Concord grape polyphenols (GP) for 13 weeks.	Concord grape polyphenols (a total of 1% GP in the diet)	C57BL/6J	GP increased the growth of *A. muciniphila* and inhibited the growth of *Firmicutes* and *Bacteroidetes.*	16S rRNA sequencing, quantitative PCR	Roopchand et al.
	2020	CRC mice received D_3_/kg supplement diet along with disease induction.	Vitamin D (0, 1500, or 3000 IU)	C57BL/6 mice	Vitamin D deficiency aggravated the deterioration of enteritis in mice, whereas vitamin supplementation regulates intestinal probiotics, particularly *akkermansia.*	16S rRNA sequencing, quantitative PCR	Zhou et al.
	2021	Mice were fed 15% or 25% anthocyanin-containing purple potato-supplemented diet for 8 weeks before treated with 2% w/v DSS for 6 days	Anthocyanin-containing purple potato (15% or 25% of diet)	C57BL/6 mice	Anthocyanin-containing purple potato	Real-time PCR	Li et al.
	2021	3% DSS + Gochujang was orally administered to rats for 7 days, followed by a 7-day recovery phase.	Gochujang (2g/kg)	SD rats	*A. muciniphila* showed no significant changes when administering DSS, but was increased when given Gochujang.	16S rRNA sequencing	Mahoro et al.
	2021	Balb/c mice were orally gavaged with hyaluronan daily for two weeks before mice were infected with *C. rodentium.*	Hyaluronan: 150 µl	Balb/c mice	Hyaluronan ttenuated Citrobacter-induced dysbiosis and increased *A. muciniphila* level.	quantitative PCR	Mao et al.
	2021	Mice were given 2.5% (wt/vol) DSS in drinking water for 7 days, and then given Epigallocatechin-3-gallate oral or rectal administration for3 days.	Epigallocatechin-3-gallate (50mg/kg body weight)	C57BL/6 J mice	Epigallocatechin-3-gallate administration attenuated colitis and promoted *A. muciniphila* abundance.	16S rRNA sequencing	Wu et al.
	2022	C57/BL-6N mice were fed high-fat diet supplemented with inulin for 8 weeks.	Inulin (10% w/w)	C57/BL-6N mice	Inulin supplementation increased A. muciniphila abundance in host gut	16S rRNA sequencing	Pérez-Monter et al.
Diets	2017	Faecal samples were collected from Egyptian and U.S. male teenagers	Mediterranean and Western diet	Egyptian and U.S. male teenagers	Egyptian teenagers who consumed Mediterranean diet observed more abundant A. muciniphila in their gut microbiota composition than their U.S. counterparts consuming Western diet	High-throughput DNA sequencing	Shankar et al.
	2018	Pathogen-free Swiss Webster mice were fed ketogenic diet for 14 days	6:1 fat: protein KD diet	Pathogen-free Swiss Webster mice	Levels of *A. muciniphila* and *Parabacteroides* were elevated	16s rDNA microbiota profiling	Olsen et al.
	2021	Mice were given KD or LCD diet for16 weeks before DSS was administered for colitis induction	KD (89% kcal fat, 10% kcal protein, <1% carbohydrate) or LCD diet (70% kcal fat, 20% kcal protein, 10% kcal carbohydrate)	C57BL/6J mice	The abundance of *A. muciniphila* was increased both in the mice gut and in mesenteric lymph nodes	16s rDNA microbiota profiling, quantitative real-time PCR	Kong et al.
	2022	Mice were fed with one of two amino acid-balanced diet recipes. After 10 days of environmental adaptation, they were given 2.5% DSS for colitis induction	Amino acid-balanced diets	C57BL/6JNifdc mice	Amino acid-balanced diet alleviated IBD symptoms and restored the level of *A. muciniphila* that was reduced during colitis devlopment	Quantitative PCR, 16S ribosomal RNA sequencing	Li et al.
Drugs	2012	Vancomycin hydrochloride was dissolved in water and given to neonatal mice pups from birth until weaning, while their mothers also received vancomycin hydrochloride (0.5 g/l) in the drinking water. Another group of mice received vancomycin hydrochloride in the water from 8 weeks of age to diabetes onset	Vancomycin hydrochloride (83mg kg^-1^ day^-1^ for pups and 0.5g/l for adult mice)	Female NOD/BomTac mice	A. muciniphila rose as the most abundant species in the host gut (>85%), while the abundance of previously dominant phylum Firmicutes and Bacteroidetes significantly decreased	16S rRNA sequencing	Hansen et al.
	2016	Rhubarb extract were supplemented into the standard diet of the mice. After 17 days of dietary treatments, mice received ethanol solution by intragastric gavage.	Rhubarb extract (30% w/v, 6 g/kg body weight)	Male C57BL/6J mice	Acute alcohol consumption led to lower relative abundance of *A. muciniphila*, which was counteracted upon rhubarb treatment.	16S rRNA sequencing, quantitative PCR	Neyrinck et al.
	2021	Metformin was given to mice with DSS-induced colitis *via* intraperitoneal injection either 3 days after or 1 day before disease induction.	Metformin(150 mg/kg)	C57BL/6 mice	Metformin ameliorated IBD symptoms and restored intestinal flora, including the abundance of *A. mucinphila* in both treatment and pretreatment group.	16S rRNA sequencing	Liu et al.
	2017	C57BL/6 mice was given Chlorogenic Acid (ChA)-containing water for 15 days and 2.5% DSS administration was given to mice for the last 8 days.	ChA (1mM)	C57BL/6 mice	*A. muciniphila* abundance was augmented by ChA in comparison to the control group.	16S rRNA sequencing	Zhang et al.
	2019	Mice were given 1 mg TNBS in 0.1 ml of 50% ethanol and resveratrol was then orally administered to the mice.	Resveratrol (100 mg/kg)	BALB/c mice	Resveratrol administration increased *A. muciniphila.*	16S rRNA sequencing	Alrafas et al.
	2019	Mice were given 3% DSS-containing drinking water for 6 days to induce colitis and then orally administered HABN for 5 days.	Hyaluronic acid-bilirubin nanomedicine (HABN) (30 mg/kg)	C57BL/6 mice	HABN significantly augmented *A. muciniphila* abundance.	quantitative PCR	Lee et al.
Herbs	2021	3% DSS was given to mice for 7 days (day 1-7) and Baitouweng decoction was administered from day 1-10.	Baitouweng decoction (5 g/kg)	Male C57BL/6J mice	Baitouweng dedoction alleviated UC symptoms and reduced the levels of pro-inflammatory cytokines. *Bacteroidetes* abundance was reduced but *A. muciniphila* was promoted.	16S rRNA sequencing, quantitative PCR	Xuan-qing.
	2021	SD rats were given 5%DSS for 7 days to induce acute experimental colitis and the administered by Jiawei Gegen Qinlian Decoction once a day for 9 days.	Jiawei Gegen Qinlian Decoction (128.65 mg/kg, 257.30 mg/kg)	SD rats	Jiawei Gegen Qinlian Decoction protects against ulcerative colitis, and regulate the gut microbiota by increasing *Akkermansia*.	16S rRNA sequencing	Li Q et al.
	2021	Rats were treated with 4% DSS for 7 days to induce UC. Zuo-Jin-Wan treatment was started 24 h after DSS administration and lasted for 6 days *via* oral gavage.	Zuo-Jin-Wan	SD rats	Zuo-Jin-Wan has shown therapeutic effects against UC *via* specific enrichment of genus *Bacteroides* and *Akkermansia.*	16S rRNA sequencing	Cai Y et al.
	2022	C57BL/6 mice were given DSS for 7 days to prepare the UC mouse model, and then were orally administered for 14 days.	Zingiber officinale-Panax ginseng herb pair	C57BL/6 mice	The abundance of Akkermansia was increased after the intervention of Zingiber officinale-Panax ginseng herb pair.	16S rRNA sequencing	Wan Y et al.

### 4.1 Supplementation with viable *A. muciniphila*

*A. muciniphila* has been isolated, identified and studied for almost 20 years, yet very few human studies on direct *A. muciniphila* supplementation are available at the current stage, especially for patients with IBD. However, several mice intervention studies have been reported and all of them showed a significant protection of *A. muciniphila* against intestinal inflammation. Bian et al. did a similar study focusing on viable *A. muciniphila*, separating their mice into three groups, namely the control group (CP), experimental group (DP) and *A. muciniphila* group (AKK). 2% (wt/vol) DSS was dissolved in the drinking water of DP and AKK group for 7 days (day 0-6) before changing back to normal water. *A. muciniphila* was administered solely to AKK group *via* oral gavage for 14 consecutive days (day -7 to day 7). Their results also support *A. muciniphila*’s modulative role on barrier function by showcasing the alleviation of DSS-induced colitis symptoms (e.g., weight loss, colon shortening) after *A. muciniphila* administration on mice (54). *A. muciniphila* improved gut microbial composition by promoting species such as *Akkermansia* and *Rikenellaceae*, although there are also some inconsistent results from other studies showing that *A. muciniphila* treatment did not change the gut microbiota profile (other than *A. muciniphila* itself) (53). Similar experimental design was adapted to test the effect of a range of *A. muciniphila* concentration and on different mice models: lower daily dose of *A. muciniphila* (1× 10^9^ - 2× 10^9^ CFU) was proved to relieve colitis in BALB/c and AAA mice, separately (45, 53).At the meantime, APOE-/- and E3L.CETP mice given Western diet witnessed a reduction in intestinal permeability and systemic inflammation after 8 weeks and 4 weeks of *A. muciniphila* supplementation, respectively, as indicated by the studies of Li et al. and Katiraei et al. (40, 51).

### 4.2 Supplementation with pasteurised or heat-killed *A. muciniphila*

In addition to viable *A. muciniphila*, pasteurised and heat-killed *A. muciniphila* as well as Amuc_1100 are now gaining increasing interests of research to test their abilities in regulating gut microflora and mucosal functions. In a study conducted by Everard et al., mice fed with control or high fat diet were treated with either *A. muciniphila* MucT (ATTC BAA-835 (2.10^8^ cfu/0.2mL) or heat-killed *A. muciniphila via* oral gavage. Mice caecal and colonic content witnessed 100-fold more viable bacteria of interest in the *A. muciniphila*-treated group (9.5 ± 1.02 log10 cells/mg of content) than their counterparts from the high fat and heat-killed bacteria group (6.8 ± 0.51 log10 cells/mg of content). Heat-killed *A. muciniphila* also lost their ability to restore colonic mucus impairments or metabolic defects triggered by high-fat diet and obesity (46). While viable *A. muciniphila* counteracted high-fat diet-induced metabolic endotoxemia and reduced mucus layer thickness, heat-killed *A. muciniphila* failed to make any significant improvements. These findings further evidenced that *A. muciniphila* has to be alive to exert its regulatory effect on the mucosal barrier. Wang et al. investigated the effects of pasteurisation-inactivated *A. muciniphila* on blunting colitis development as well as the associated tumorigenesis. 1.5×108 CFU pasteurised *A. muciniphila* was administered to colitis or colorectal cancer mice models established using DSS and azoxymethane. qPCR, 16S rRNA sequencing, and flow cytometry results confirmed that pasteurised *A. muciniphila* positively influenced hosts’ immune system by reducing infiltrating macrophages, decreased proportions of CD8^+^ cytotoxic T lymphocytes in the colon and relieved ameliorated colitis in mice. These data indicated that pasteurised *A. muciniphila* provides potential prevention and treatment strategies for colitis as well as other IBDs (23).

### 4.3 The outer membrane protein Amuc_1100*, A. muciniphila*-derived extracellular vesicles and *A. muciniphila*-secreted protein P9

In addition to viable or heat-killed *A. muciniphila*, emerging evidence shows that the outer membrane protein Amuc_1100, bacteria-derived extracellular vesicle (AmEVs) and *A. muciniphila*-secreted protein (P9) also play important roles in preventing and/or treating IBD. To test the effects of Amuc_1100 on IBD, Wang et al. gave DSS-induced colitis model mice oral administration of 3μg Amuc_1100 from 2 weeks before DSS treatment to sacrifice. Analysis on stool and colon samples showed that significantly alleviated disease activity index and attenuated colonic histological injuries and inflammations. Interestingly, Amuc_1100 also downregulated pro-inflammatory cytokines including TNF-α and IFN-γ, suggesting an anti-inflammatory effect in the host gut (Wang et al., 2019). Extracellular vesicles are spherical structures consisting of lipid bilayers. They are released by bacteria and are capable of entering the host’s systemic circulation and even delivering their components into host cells. Thus, these structures can induce immunological and metabolic responses, and consequently interact with gut microbiota or the host (48, 57). Kang et al. reported that oral administration of AmEVs ameliorated symptoms of colitis including weight loss, colon shortening and inflammatory cell infiltration of gut barrier (57). *In vitro* experiments conducted on colon carcinoma cells showed that AmEVs application can reduce the secretion of pro-inflammatory cytokine IL-6 from colon epithelial cells, providing a possible explanation for AmEVs’ mechanism in regulating intestinal immunity and homeostasis. Their findings were supported by Chelakkot and colleagues, who reported that AmEVs improved gut barrier integrity and reduced intestinal epithelial layer damage from high fat diet (48). Despite the effects of AmEVs, it remains unclear whether AmEVs contain Amuc_1100 or other confounding factors that may exert an effect on gut immunity and metabolism. Therefore, more studies are required to investigate the properties of these bacterial structures. P9 is an 84kDa, glucagon-like peptide 1 (GLP-1)-inducing protein produced by *A. muciniphila*. Yoon et al. administered 100μg P9 daily to HFD-fed mice for 8 weeks and concluded that the administration of P9 prevented mice against obesity and improved glucose tolerance by regulating GLP-1 secretion (58). Several studies have shown that L cell-secreted GLP-1 has been associated with reduced levels of pro-inflammatory cytokine IL-1β, increased mucus-secreting goblet cells and improved gut epithelial architecture. Therefore, it is thus reasonable to postulate that P9 may alleviate the inflammation and intestinal injuries in IBD by modulating GLP-1 expression (59). However, a recent study also showed that P9 induced the expression of IL-6 in macrophages, suggesting its pro-inflammatory role in the occurrence and development of intestinal inflammation. On the whole, research for the properties and effects of this protein is very scarce, and further *in vitro* studies as well as mice experiments are needed for researchers to fully understand its mechanisms in regulating gut homeostasis and potentially alleviating IBD.

#### 4.3.1 Probiotics

Administering several strains of probiotic treatments has been proved to have a promoting effect on *A. muciniphila* abundance. In addition to direct administration of *A. muciniphila*, a single probiotic treatment with *Lactobacillus pentosus* on mice with DSS-induced colitis was proved to increase the level of *A.muciniphila* abundance. Inflammatory cell infiltration and colonic injuries due to colitis were also alleviated post-treatment, thus inhibiting the development of the disease (60). Like *Lactobacillu pentosus*, other members of the *Lactobacillu* species have been widely researched as promising probiotic to regulate gut microbiota and promote intestinal health, although not all strains have been directly linked with promoting *A. muciniphila* or improving IBD. *Lactobacillus plantarum*, strain WCFS1, for example, had been proved to enhance TLR2 signalling activities, occluding expression, and subsequently benefit the mucosal barrier of the gut (61). It is thus reasonable to postulate that these modulations may associate with an increase in *A. muciniphila* level. A recent study supported this hypothesis by showing that, four weeks of 3 × 10^10^ CFU/day *Lactobacillus* plantarum administration to healthy triathletes significantly increased the abundance of *A. muciniphila* (62). Several mixtures of probiotics have also been found to be able to rebuild the gut microbiota structure and consequently improve IBD. *Bifidobacterium infantis*, *Lactobacillus acidophilus*, Enterococcus faecalis and *Bacillus cereus* collectively exert a promoting effect on *A. muciniphila*, almost restoring the gut microflora structure to normal state (63). Another study showed that administering *Bifidobacterium animalis subsp. lactis* LMG P-28149 or a mixture with *Lactobacillus rhamnosus* (Mix) for 14 weeks can restore the composition of host gut microbiota and significantly promote *A. muciniphila* abundance. Moreover, the Mix enhanced the production of SCFAs (e.g., butyrate and acetate), which could potentially promote mucin growth and consequently feedforward to *A. muciniphila* level increase, bringing beneficial changes to the host intestinal immunity. It is worth noticing that the protective effect of the Mix is likely resulted from *Bifidobacterium animalis subsp. lactis*, as administering *Lactobacillus rhamnosus* in its own decreased the abundance of *A. muciniphila* (64).

#### 4.3.2 Prebiotics

Prebiotic are dietary substances that stimulates growth or activity of specific species in the gut microbiota that give rise to beneficial effects to the host (65). Bu et al. tested the effect of 7-day prebiotics administration on host mice gut microbiota. Interestingly, mice treated with the total flavone of *Abelmoschus Manihot* (TFA), a prebiotic of *A. muciniphila*, showed a direct promoting effect on *A. muciniphila* in a dose-dependent manner within certain range. The inhibiting effect on pro-inflammatory cytokines was also boosted with increased concentration of TFA, which subsequently contributed to improved gut barrier permeability and immunity. Notably, experimental colitis reduced the mRNA expression of KLF4, MUC2 and ZO-1in their experiment. KLF4 is a transcription factor associated with intestinal cell proliferation (especially goblet cells). Mucin MUC2 blcoks pathogens in the gut lumen, and deficiency in MUC2 leads to a destruction of symbiotic bacterial habitat. ZO-1 is involved in maintain the integrity of gut barrier and contributes to the recruitment of other component during the assembly of tight junctions. KLF4, MUC2 and ZO-1 expression were all improved after TFA treatment (66). Hyaluronan, a glycosaminoglycan polymer, has also shown prebiotic properties facilitating the maintenance of gut homeostasis and ameliorating IBD symptoms. Our previous studies showed that hyaluronan attenuated *C. rodentium*-induced colitis by boosting *A. muciniphila* abundance, promoting the expression of antimicrobial peptides, restoring the gut microbiota composition, and consecutively protecting the host against *C. rodentium* infection. What’s more, dietary supplements of prebiotic were also widely investigated for their effects on promoting *A. muciniphila*. For example, vitamin D3 supplementation promoted *A. muciniphila* (among other beneficial probiotics such as *Bifidobacterium*) in healthy human participants. Zhou and colleagues found that the level of vitamin D3 was significantly reduced in DSS- and Azoxymethane- induced CRC model mice and in CRC patients. While vitamin D deficiency is associated with aggravated enteritis deterioration, supplementation of this substance restores the number of *A. muciniphila* and boosts *A. muciniphila*-mediated colon barrier integrity (35). Intake of inulin, a fermentable fructo-oligosaccharide, has been associated with increased level of *A. muciniphila* in both healthy and obese participants (67), while giving mice inulin supplements in addition to a fat-enriched diet also increased the level of A. muciniphila in the host gut (68). Another recent study revealed that anthocyanin-containing purple potatoes in the diet alleviated DSS-induced colitis. Li et al. fed their colitis model mice a diet supplemented with 15% or 25% baked and freeze-dried purple potato powder and observed very intriguing outcomes: adding either proportion of purple potato powder suppressed colitis-induced body weight loss and inflammation. Pro-inflammatory interleukins IL-6 and IL-17 were downregulated and pathogenic bacteria such as *Enterobacteriaceae* abundance were reduced. 25% purple potato powder diet also elevated *A. muciniphila* abundance by four folds (69). Administrations of dietary polyphenols, natural antioxidants that sometimes possess antimicrobial properties, gave inconsistent results in mice studies. Concord grape polyphenols and epigallocatechin-3-gallate, a bioactive polyphenol constituent in green tea promoted *A. muciniphila* abundance in mice (70, 71). Wu et al. gave DSS-induced colitis mice 3 days of oral or rectal administration of epigallocatechin-3-gallate following disease induction, showing that this green tea-derived polyphenol attenuated colitis symptoms such as colon shortening. However, supplementing 4% green tea powder to HFD-fed mice did not induce any significant changes in *A. muciniphila* abundance (72). These results suggest that the properties of dietary polyphenols may be dependent on their sources and dose (73).

#### 4.3.3 Diets

Difference in dietary habits may also influence microbiota and subsequently colonic mucosal integrity. Ketogenic diets (KD), characterised by high-fat and low-carbonhydrate, was initially used in treating epilepsy, but has now shown increasing potential in treating a wide range of other diseases. Recent studies suggest the possibility that the therapeutic potential of KD may attributed by its impact on the gut microbiota. Olson and colleagues gave healthy specific pathogen-free Swiss Webster mice a 6:1 fat: protein KD diet and found that these mice showed significant increase in the relative abundances of *A. muciniphila* (74). Kong et al. confirmed the effect of KD on alleviating IBD and found that KD (89% kcal fat, 10% kcal protein and <1% carbohydrate) enriched *A. muciniphila* abundance in mice gut and mesenteric lymph nodes, regardless of whether these mice have colitis. *A. muciniphila* dominated the gut microbiota in KD-treated colitis model mice, while colitis symptoms were also alleviated. In addition to changes in the gut microbiota composition, metabolites such as L-asparagine and glycine observed an increase, while the amount of ILC3 cell production was reduced. On the other hand, low carbohydrate diet (LCD, 70% kcal fat, 20% kcal protein, 10% kcal carbohydrate) decreased *A. muciniphila* level in the host gut and aggravated intestinal inflammation by promoting the production of pro-inflammatory IL-18 (75). High-fat diets had been previously associated with impairments of intestinal function, and it is yet unclear how KD exerts a protective effect on the intestinal barrier. However, it is postulated that increased L-asparagine resulting from KD could inhibit intestinal inflammation and protect the intestinal barrier from LPS-induced damages (75, 76). Mediterranean diet, characterised by high monosaturated fatty acids, fibre, whole grain, beans, nuts and low consumption of meats and sweets, is considered to have beneficial effects on immune and metabolic systems. In contrast, Western diet consumed by most industrialised countries is low in fruits and vegetable, but high in fat (especially animal fats) and sugar. Shankar et al. compared the distal gut microbiota composition in Egyptian (Mediterranean diet) and U.S. (Western diet) teenagers. They found that the abundance of *A. muciniphila* is significantly higher in Egyptian teenagers, while the gut of U.S. teenagers had relatively more *Bacteroides*, which might indicate an adaptation to elevated inflammatory levels in Western populations (77). The effects of dietary amino acid composition on treating IBD were also investigated in prior studies. Li et al. developed two recipes (Recipe 1 and 2, or R1 and R2) for amino acid-balanced diets based on grains (rice, millet, quinoa and mung bean), and beta-glucan and fructooligosaccharides were adopted for better functional and sensory qualities. Feeding DSS-induced colitis model mice with amino acid-balanced diets ameliorated IBD symptoms, and particularly, R1 (amino acid score: 86.64 ± 0.45, chemical scores: 104.36 ± 3, essential amino acid index: 61.02 ± 0.9) diet restored the decreased abundance of *A. muciniphila* (78).

#### 4.3.4 Drugs

Given the close relationship of *A. muciniphila* with gut microbiota homeostasis, more and more researchers are now seeing this bacterium as a potential target for pharmaceutical intervention. Postulated treatments to modify the microbial environment of the host gut range from traditional antimicrobics to the relative novel nanomedicines (79–81). One of the most studied drugs known to promote *A. muciniphila* is metformin, a drug that has been used in type 2 diabetes treatment. While its therapeutic mechanisms are not yet fully understood, recent studies have revealed that this drug is involved in the modulation of gut microbiota, which consecutively contributes to reducing inflammation or obesity. De la Cuesta-Zuluaga et al. examined 28 type-2 diabetic patients- 14 taking metformin whilst others did not- to investigate the gut microbiota composition in their faecal samples (82). Their research has shown that *A. muciniphila* was more abundant in diabetic patients taking metformin than either diabetic participants who were not taking the drug, or their healthy counterparts. C57BL/6 mice experiments were consistent with the above clinical studies: metformin was given to mice with DSS-induced colitis *via* intraperitoneal injection (150ng/kg/day) either 3 days after metformin treatment, or MD group) or 1 day before (pre-treatment, or p group) disease induction. Both MD and p group significantly reduced inflammation, inhibited colon shortening and increased *A. muciniphila* (83). Metformin treatment *via* 4-10 weeks of oral gavage (100-300 mg/bw) showed a similar result in terms of promoting *A. muciniphila* (38). However, debate remains regarding whether the beneficial effects in these studies resulted from metformin or unknown confounding factors: an antibiotic combination (carbenicillin, metronidazole, neomycin, vancomycin) pre-treatment abolished the antidiabetic effect (38). The interaction between metformin and antibiotic drugs seem to suggest that the effect of metformin is subjected to gut microflora activities. Metformin also increases the number of goblet cells in the mice ileum, which may have a bidirectional boosting effect with *A. muciniphila*.

In addition to metformin, antibiotic treatments also often lead to alterations in microbial diversity. A recent study revealed that vancomycin treatment on mice promoted *A. muciniphila* as the most abundant species in the host gut (>85%), while the abundance of previously dominant phylum Firmicutes and Bacteroidetes significantly decreased (80). Dubourg et al. conducted a study on two patients receiving broad-spectrum antibiotic treatment, which supported the above findings: patient A was receiving a combination of doxycycline, hydroxychloroquine, piperacillin/tazobactam and teicoplanin at the time of stool collection, while patient B received impinem. Surprisingly, despite the fact that *A. muciniphila* is susceptible to imipenem and piperacillin, it was found to make up > 40% of the total gut microbiota (44.91% in patient A and 84.64% in patient B) without inducing any significant gastrointestinal disorders (84).

#### 4.3.5 Herbs

Complementary and alternative medicines, especially herbs, have increasingly been used in the treatment of UC patients in recent years, and has been shown a fundamental role in regulating the gut microbiota during UC management (85). Zingiber officinale and Panax ginseng have been used together to clinically treat ulcerative colitis with synergistic effects for thousands of years, whose mechanism was related to regulating gut microbiota according to Wan et al.’s study showing that the relative abundance of beneficial bacteria (such as *Akkermansia*) was significantly increased and the abundance of pathogenic bacteria was markedly decreased after the intervention of Zingiber officinale-Panax ginseng herb pair (86). Some herbal prescriptions from traditional Asian medicines were also researched for their therapeutic effects on IBD. Baitouweng decoction is a prescription in traditional Chinese medicine that consists of four herbs (i.e., Radix pulsatilla, Cortex phellodendri, Rhizoma coptidis and Cortex fraxini) and it is used to treat UC. Results from a DSS-induced colitis model mice study showed that Baitouweng decoction reduced the production of several pro-inflammatory cytokines. Furthermore, *Bacteroidetes* was reduced after Baitouweng dedoction intragastrical administration, whereas *A. muciniphila* abundance was promoted (87). Similar to this study, the results from Li et al. showed that the main active components of Jiawei Gegen Qinlian decoction protected against ulcerative colitis under different dietary environments in a gut microbiota-dependent manner, especially regulating the gut microbiota by increasing the abundance of *Akkermansia* and decreasing the colonisation of *Escherichia-Shigella* (88). Similar to this study, a study from Cai et al. also showed a specific enrichment of genus *Akkermansia* in DSS-induced intestinal inflammation. All these results showed a significant role of herbs in the enrichment of *A. muciniphila* abundance (89).

## 5 Conclusions and perspectives

The current evidence has demonstrated its potential protective effect of *A. muciniphila* in the process and development of intestinal inflammation. Abnormal reduction in abundance of *A. muciniphila* maybe a hallmark of IBD, which is associated with gut dysbiosis, decreased mucosal barrier function and altered immune response. However, the role of commensal *A. muciniphila* in IBD is still controversial, and even plays a pro-inflammatory role in the occurrence and development of intestinal inflammation, which might be due to difference in genotypes of host, strain specificity of *A. muciniphila* and the coexistence of enteropathogens. Therefore, we should always hold a reasonable dose of expectation and skepticism in terms of the overwhelming “good effects” of *A. muciniphila* in health and disease states.

Nevertheless, *A. muciniphila* -based and -targeted therapies are now considered a valuable therapeutic approach to treat IBD patients. However, how to selectively enrich the growth and colonisation of *A. muciniphila* in the host intestine with direct or indirect interventions have not been clarified. This review reveals an interesting phenomenon that not only viable *A. muciniphila*, but also pasteurised *A. muciniphila*, and its ingredients such as AmEVs, Amuc_1100 and P9 also can alleviate intestinal inflammation, suggesting a therapeutic potential of *A. muciniphila* in the treatment of IBD. But there is still a long way to go to develop the clinically available products. Firstly, the strict anaerobic characteristics of *A. muciniphila* make it difficult to isolate, purify and preserve, so there is an urgent need for the development of novel strategies for the industrial production of *A. muciniphila*. Secondly, considering the controversial role of *A. muciniphila*, host genotypes and intestinal microenvironment, especially the coexistence of pathogenic bacteria in gut, have to be comprehensively and fully taken into consideration before its clinical application. Thirdly, safety issues of *A. muciniphila* needs to be further evaluated in the treatment of IBD. Up to now, there is still a lack of clinical studies in treating of IBD with *A. muciniphila* alone, which means that the effective dose, optimal dose, treatment course and adverse effects are not clarified and need to be further determined.

**Figure 2:**
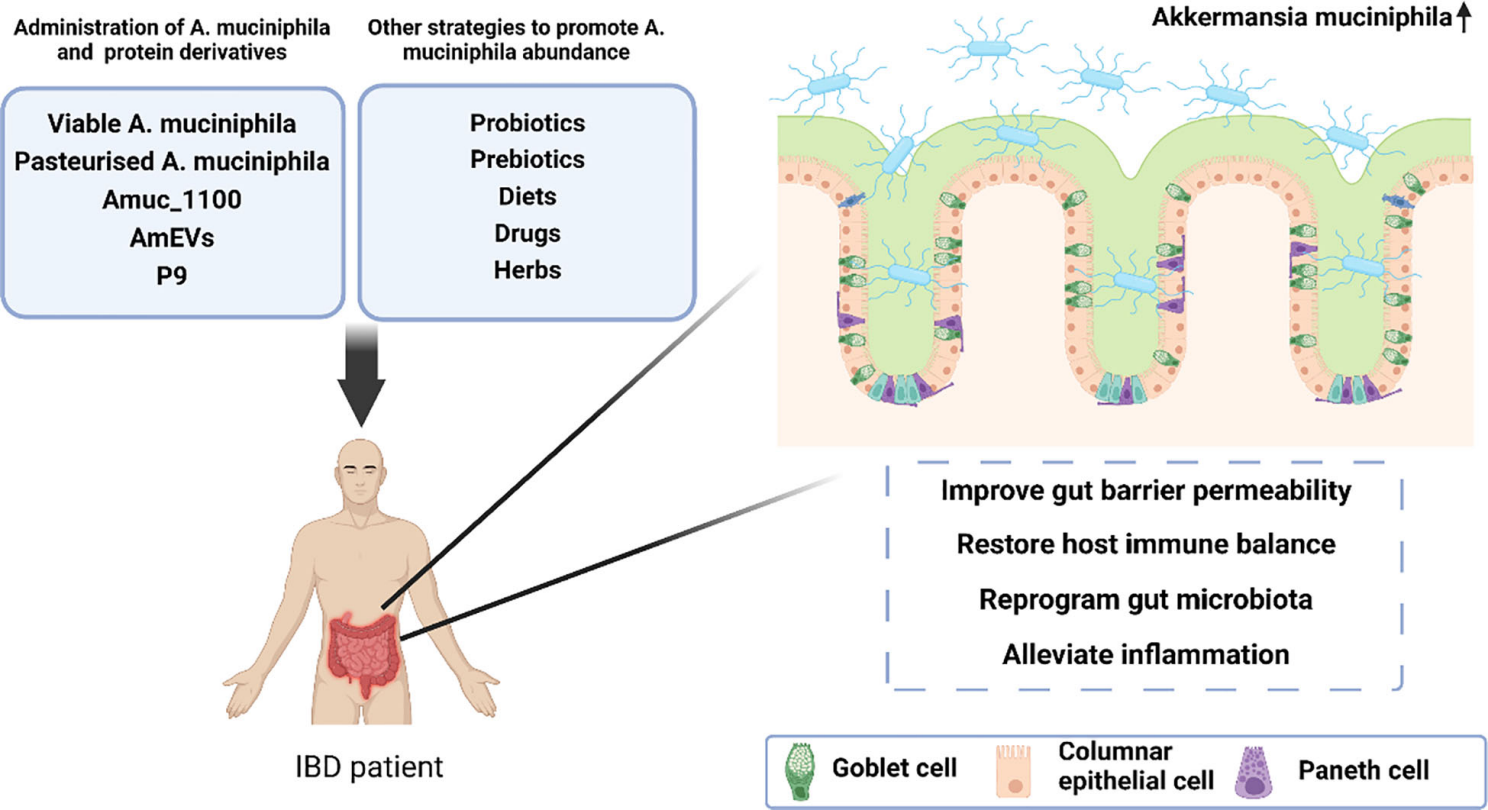
A proposed model illustrating the protective effect of *A. muciniphila*-targeted and -based therapies in the treatment of inflammatory bowel disease.

More importantly, because of the natural characteristics individual strains grow, we believe that it is more promising weapon for the treatment of IBD to improve the intestinal microenvironment of *A. muciniphila* than to simply increase the number of individual bacteria itself. Therefore, it has become a research hotspot in recent years to improve intestinal microenvironment and indirectly promote the colonisation of *A. muciniphila* through the supplementation of other probiotic or prebiotics, natural diets, drugs, and herbs, and have achieved good progress (**Figure 2**), which holds promise for *A. muciniphila* -based and -targeted therapies in the treatment of IBD.

## Author contributions

TM and JL conceived and designed the study. MZ, RH and YY collect literatures, organized documents, and wrote the first draft of the manuscript. YX, WZ, ZS and YL participated in editing of specific paragraphs and figures. TM supervised and critically revised the manuscript. All authors read and approved the manuscript. All authors contributed to the article and approved the submitted version.
